# Pfetin as a Risk Factor of Recurrence in Gastrointestinal Stromal Tumors

**DOI:** 10.1155/2014/651935

**Published:** 2014-05-26

**Authors:** Hajime Orita, Tomoaki Ito, Tomoyuki Kushida, Mutsumi Sakurada, Hiroshi Maekawa, Ryo Wada, Yoshiyuki Suehara, Daisuke Kubota, Koichi Sato

**Affiliations:** ^1^Department of Surgery, Juntendo Shizuoka Hospital, Shizuoka, Japan; ^2^Department of Pathology, Juntendo Shizuoka Hospital, Shizuoka, Japan; ^3^Proteome Bioinformatics Project, National Cancer Center Research Institute, Japan

## Abstract

*Background*. Despite complete resection of gastrointestinal stromal tumors (GIST), recurrent and/or metastatic disease occurs, often depending on the grade of malignancy. As such, markers are needed that accurately predict patients at high risk for recurrence. Previously our group reported Pfetin as a prognostic biomarker for GIST. In order to create an approach for predicting risk of recurrence, we incorporated Pfetin expression with clinicopathological data to produce a predictive model. *Object*. Forty-five patients with localized primary GIST were treated with complete gross surgical resection surgically at our institution between 1995 and 2010 were included. The majority of tumors originated in the stomach (38 cases), as well as small intestine (6 cases) and rectum (1 case). *Method*. (1) We performed retrospective analysis of the connection between Pfetin expression, clinicopathological data, and incidences of recurrence, using bivariate and multivariate analyses. (2) The reactivity of the monoclonal antibody against Pfetin was examined by immunohistochemistry. *Pfetin*. We have reported Pfetin, identified microarray technology, and compared between statistically different GISTs for good and poor prognoses and for prognostic marker. *Results*. There were 7 cases of recurrences. (1) By univariate analysis, tumor size, mitoses, exposure to abdominal cavity, and complete tumor removal predicted risk of recurrence. (2) Pfetin-negative cases were significantly related to recurrence (*P* = 0.002). *Conclusions*. This analysis demonstrates that lack of Pfetin expression is an additional predictor of recurrence in resected GIST. Further study may determine the role of this variable added to the current predictive model for selection of adjuvant therapy.

## 1. Background

Gastrointestinal stromal tumor (GIST) is the most common mesenchymal tumor of the digestive tract. The precise cellular origin of GIST recently has been proposed to be the interstitial cell of Cajal, an intestinal pacemaker cell [1–3].

Although it comprises a low incidence (2/100,000 people/year) in Japan, their rates of recurrence and mortality remain high (30–40%) [4, 5].

These tumors are almost 90% associated with mutations of c-KIT [6, 7]. And recently c-kit negative cases have been identified as harbor activating mutations of platelet-derived growth factor receptor alpha (PDGFRA).

Most frequently, GISTs are located in the stomach (more than 60%) and the small bowel (30%) but can arise anywhere from the esophagus to the rectum and in the omentum and peritoneum [8, 9].

Before imatinib mesylate (inhibitor of tyrosine kinase) was developed, there were no effective treatments. GISTs uniquely metastasize by hematogenous spread and peritoneal seeding. It is most frequently recurrent in the liver, omentum, and peritoneum [8, 10]. The 5-year survival rate was 11–30%. In 2001, the first reported case of imatinib mesylate yielding striking effects in the treatment of GISTs was reported [11]. Imatinib dramatically changed the mean survival time of GIST (from 32–39 to 58 months) [8].

Despite complete resection, GISTs sometimes recur or metastasize, according to the degree of the malignancy. Viewed in this light, there are many arguments for adjuvant therapy. Treatment for recurrent and/or metastatic GIST with imatinib has shown effectiveness. The recent Z9001 trial in the USA that tested 1 year of adjuvant treatment by imatinib reported reduction of the recurrences and metastases (Lancet) [8]. As such, the standard of care for high-risk patients after complete resection is one year of adjuvant therapy.

There are also risks associated with imatinib treatment which include costs and drug resistant from long time treatment by imatinib. One of the imatinib induced complications is the GI bleeding, such as GAVE (define). It can be due to the presence of residual tumor or to other less common etiologies [12, 13]. A multitude of dermatological toxicities also occurs, from various acute rashes to Steven-Johnson syndrome [14].

As with any therapy, balancing risks and benefits is paramount. Thus, it is necessary to diagnose the high-risk group for recurrences and/or metastases accurately. A sensitive and specific marker is sought to select patients at high risk for recurrence. Previously our group has reported Pfetin as a prognostic biomarker for GIST, identified using a proteomic approach [15]. Pfetin was originally cloned as a gene highly expressed in the fetal cochlea and brain [16]. Pfetin is also a member of KCTD family [16] and contributes to carcinogenesis and cancer progression.

The aim of this study was to determine the role of Pfetin expression in predicting the incidence of recurrence, in particular related to incorporation of this marker into the current model.

## 2. Patients

We examined the primary tumor tissues of 45 GIST patients who underwent surgery with complete (R0) resection at our institution between 1995 and 2011. Tumors originated in the stomach (37 cases), duodenum (1 case), small intestine (6 cases), and rectum (1 case). There were 7 recurrences. Diagnosis was based on the World Health Organization classification system for soft-tissue tumors: tumor size, presence of necrosis, differentiation, mitotic rate, MIB-1 index, and presence of epithelioid cells. C-kit expression in all GIST samples was confirmed using immunohistochemical staining (CD117 antibody, DAKO Japan Corp., Tokyo, Japan). Clinicopathological features of the 45 GIST patients are listed in Table 1.

## 3. Pathologic Analysis

All tumors included in the study were rereviewed by one pathologist, and the diagnosis of GIST was confirmed by positive staining for KIT (CD117) protein, as previously described. Tumor morphology was classified as predominantly epithelioid or spindle-shaped. Mitotic rate was determined by counting the number of mitotic figures per 50 high power fields (HPF) and categorized as <5, 5–10, or _10. Mib-1.

## 4. Immunohistochemistry

Pfetin expression was examined immunohistochemically using paraffin-embedded tissues. 4-mm-thick tissue sections were autoclaved in 10 mmol/L citrate buffer (pH 6.0) at 1218°C for 30 min and incubated with the antibody against Pfetin (1 : 1000 dilution). Immunostaining was carried out according to the streptavidin-biotin peroxidase method using the Strept ABC complex/horseradish peroxidase kit (DAKO). More than 20% of tumor cells were stained with the anti-Pfetin antibody which was considered to be Pfetin positive.

In most cases, the difference was quite obvious and the two reviewers concurred with the results.

## 5. Statistical Analysis

1. We performed retrospective analysis of the connection between clinicopathological data and incidences of recurrent, using bivariate and multivariate analyses.
2. All statistical analyses were carried out using the Chi2 test to assess the relationships between Pfetin expression and clinicopathological factors. The tumor-specific and disease-free survivals were calculated from the initial resection of the primary tumor until first evidence of metastasis and recurrence, respectively. All time-to-event endpoints were computed by the Kaplan-Meier method. Calculations were carried out using the SPSS software statistical package (SPSS Japan, Inc., Tokyo, Japan).

## 6. Results

### 6.1. Clinical Features

The median age of the population was 66.2 years (range: 38–87) and there were 24 (53.3%) males. Tumor locations included the stomach in 42 (82%), small bowel in 6 (13.3%), duodenum in 1 (2%), and rectum in 1 (2%) (Table 1). The median tumor size was 5.76 (0.6–15) cm. All patients had complete resections. Seven cases had recurrence and/or metastasis after resection of the primary tumor. Six cases were gastric cases (14%) and 2 cases were intestinal cases (25%).

*(1) Tumor Size and Mitosis are High-Risk Markers for GIST*. On univariate analysis, tumor size and mitoses were significantly correlated with recurrence (Table 1). These factors were a significant correlation with Fletcher's risk classification. These data led Fletcher's classification to be a good correlation with recurrences. All 7 cases of recurrence were grouped as high risk according to Fletcher's classification.

This demonstrated high accuracy. Dissemination and invasion also correlated with recurrence (Table 2).

*(2) Pfetin-Negative Cases Were Significantly Related to Recurrence*. Thirteen cases were Pfetin negative and 5/13 of these cases recurred (see Table 3). (*P* = 0.002). Clinicopathologically, Pfetin correlated with mitoses (*P* = 0.024).

These data were consistent with those in our previous study in which Pfetin expression was strongly correlated with recurrence and/or metastasis of GIST patients. Pfetin expression was strongly correlated with the prognostic value of GIST patients.

**Figure 1:**
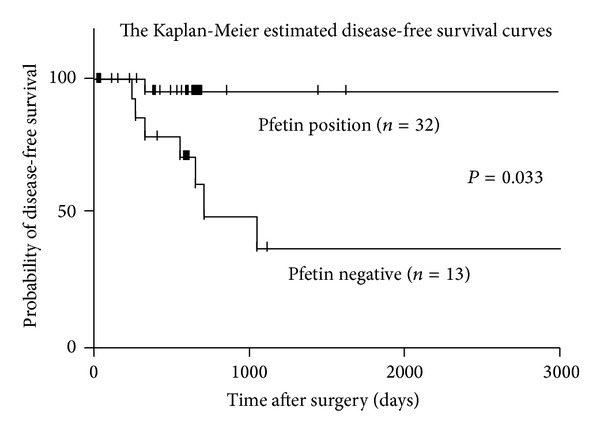
The Kaplan-Meier estimated disease-free survival curves. Pfetin expression was strongly correlated with the prognostic value of GIST patients.

The Kaplan-Meier estimated disease-free survival curves revealed Pfetin expression to correlate significantly and inversely with recurrence. (Figure 1). Disease-free survival was dramatically longer in Pfetin-positive than in Pfetin-negative cases; Pfetin expression was strongly correlated with the prognostic value of GIST patients.

## 7. Discussion

Imatinib remains the standard of care for adjuvant therapy [10, 17, 18] after surgical resection for primary, localized GIST. Given significant recurrence, despite adjuvant imatinib, we need accurate predictive models for recurrence to guide adjuvant treatment. Current risk classification systems assist in determining the risk of disease recurrence in individual patients with GIST, so disease management can be personalized. However, risks of imatinib including cost, severe side effects, and inconvenience must be considered. Additional prognostic markers are urgently needed to improve decision-making regarding adjuvant therapy for these patients.

The current standard, NIH consensus classification system for GIST, classified patients into risk groups on the basis of tumor size and mitotic index. Subsequently, Mietteinen modified this into the pathology risk stratification system by including tumor location and histology. Gold et al. reported the importance of intestinal location [19, 20] showing that intestinal GISTs are more likely to recur those gastric locations.

Nomograms created by DeMatteo presented risk of recurrence as percentages on a continuous scale. The goal of our study was to consider the impact of the addition of Pfetin level on these models.

In this study, Pfetin expression also correlated with outcome. This protein was discovered by microarray analysis using proteomic technology in Professor Kondo's lab. Pfetin's exact role remains to be determined.

Regardless, in this study test set, we showed an inverse relationship between Pfetin expression and risk of recurrence. Future testing in a validation set is planned. If this predictive relationship is confirmed, one might consider including it in a revised model.

## 8. Conclusion

Pfetin is an independent predictor of recurrence/metastasis for completely resected primary, localized GIST. Further investigation of this role is planned.

## Figures and Tables

**Table 1:** Clinicopathological features of the 45 gastrointestinal stromal tumors (GIST) cases.

		Cases	Recurrence	(%)	*P* value
Sex	Male	24	3	12.5	0.613
	Female	21	4	19.0	

Age	38~87 years old				0.771
	(mean 66.2)				

Location	Stomach	37	5	14.0	0.635
	Intestine	6	2	25	
	Duodenum	1	0	0	
	Rectum	1	0	0	

Tumor size	<5 cm	25	0	0	*P* < 0.001
	5~10 cm	10	4	40	
	>10 cm	10	3	30	

Mitosis	<5/50 HPF	32	2	6.3	*P* = 0.035
	>5/50 HPF	13	5	38.5	

MIB-1 index	≧10%	6	1	16.7	0.963
	<10%	39	6	15.4	

Tumor size and mitosis have significant correlations with recurrence. These factors are also Fletcher's agents. These data lead Fletcher's classification to be good collation to recurrences.

**Table 2:** Clinical malignant factors of the 45 gastrointestinal stromal tumors (GIST) cases.

		Cases	Recurrence	(%)	*P* value
Dissemination (tumor explosion)	Positive	9	4	44.4	0.088
	Negative	36	3	8.3	

Invasion (other organs)	Positive	4	3	75	0.094
	Negative	41	4	9.8	

Damage on operation	Positive	2	2	100	0.287
	Negative	43	5	11.6	

Tumor necrosis	Positive	8	1	12.5	0.866
	Negative	37	6	16.2	

In clinical malignant factors, dissemination and invasion have a tendency for recurrence.

**Table 3:** Pfetin expression.

		Recurrence		*P* value
		(+)	(−)	
Pfetin	Positive	2	30	0.002
	Negative	5	8	

Pfetin negative cases were significantly related to recurrence (*P* = 0.002).
